# Application of Parallel Reaction Monitoring to the Development and Validation of a Quantitative Assay for ST-246 in Human Plasma

**DOI:** 10.3390/ijms23148021

**Published:** 2022-07-21

**Authors:** Alexander A. Chernonosov, Galina A. Oleinik, Vladimir V. Koval

**Affiliations:** 1 Institute of Chemical Biology and Fundamental Medicine SB RAS, Lavrentiev Ave. 8, 630090 Novosibirsk, Russia; zakabluk@niboch.nsc.ru (G.A.O.); koval@niboch.nsc.ru (V.V.K.); 2 Department of Natural Sciences, Novosibirsk State University, Pirogova Str. 2, 630090 Novosibirsk, Russia

**Keywords:** blood plasma, tecovirimat, high-resolution mass spectrometry, LC-HRMS, PRM

## Abstract

In this work, we developed and validated a robust and sensitive method of liquid chromatography with high-resolution mass spectrometry in parallel reaction monitoring (PRM) mode for ST-246 (tecovirimat) quantification in human blood plasma. The method was compared with the multiple reaction monitoring (MRM) technique and showed better selectivity and similar sensitivity in a wider concentration range (10–5000 ng/mL). Within this range, intra- and interday variability of precision and accuracy were within acceptable ranges in accordance with the European Medicines Agency guidelines, and recovery was 87.9–100.6%. Samples were stable at 4 °C within 48 h and at −20 °C up to 3 months. The recovery and matrix effects in the proposed HRMS method were about 5% higher than those reported for the MRM method, but the PRM method showed better accuracy with comparable precision. It was found that the ST-246 concentration shown by the PRM method is approximately 24% higher than the output of the MRM one. Nonetheless, the high selectivity with similar sensitivity, as compared with traditional MRM methods, makes the proposed approach attractive for research and clinical use.

## 1. Introduction

Smallpox is a highly contagious disease; it is only slightly contagious as compared to measles and chickenpox. According to the materials of the Smallpox Eradication Program, on average, one patient could infect five people, but often, up to 20–35 infected people resulted from one person [1,2]. To defeat such a dangerous disease, in 1967, a 10-year campaign was carried out all over the world to eradicate smallpox through mass vaccination of the population. Since 1977, there has not been a single case of variola virus disease, and in 1980, the World Health Organization declared the world free of smallpox and recommended that the vaccination be discontinued [3].

Nevertheless, as a result of the widespread cessation of smallpox immunization, the proportion of the population susceptible to variola virus and other related orthopoxviruses pathogenic to humans is constantly increasing. This is confirmed by an increasing number of outbreaks of orthopoxvirus infections (in humans) caused by such viruses as variola virus, monkeypox virus, cowpox virus, and vaccinia virus [4]. In addition, vaccination has provided only protection against the disease for 3–5 years, with a subsequent decrease in protection effectiveness [5]. Therefore, even vaccinated people are currently at risk of contracting smallpox. Up to 30% of unvaccinated and 1% of vaccinated people may die if they get infected with “typical” smallpox. Due to its contagiousness, a very small dose is required for infectivity [1], and the “attack rate” or the probability that a person will contract the disease via contact with an infected person ranges from 26% to 90% [6]. Additionally, the smallpox virus is reported to be a possible biological weapon because its genome—allowing genetic manipulations—has been published [7]. The public scientific literature contains relatively simple and inexpensive methods for growing, cleaning, and genetically engineering the smallpox virus [8]. Besides, poxvirus virulence can be enhanced by the insertion of cytokine genes such as IL-4, although this has been demonstrated only in mice [9].

Until recently, there have been no effective drugs for smallpox. Nonetheless, in July 2018, the US Food and Drug Administration (FDA) registered the first smallpox drug, tecovirimat (TPOXX). This drug was created on the basis of chemical compound ST-246 [10]. An analogue of tecovirimat, a new chemical compound (NIOCH-14) has been obtained, which has a comparable activity against orthopoxviruses [11]. This compound is a prodrug of tecovirimat and is converted into it when ingested.

Due to the novelty of tecovirimat and NIOCH-14, a series of clinical, preclinical, toxicological, and other studies are required. For these purposes, developed and validated methods for tecovirimat quantitation in human plasma are required. During ST-246 investigation, some research groups have developed a quantitative assay for tecovirimat on the basis of multiple reaction monitoring (MRM) mode, predominantly for monkey, nonhuman-primate, and rabbit plasma [12,13]. In humans, the newly developed method has a high limit of quantitation (LOQ), 50 ng/mL [13,14,15], and only recently was a validated MRM method published with the calibration range of 10–2500 ng/mL [16]. There are no studies on the development and validation of such an assay based on high-resolution mass spectrometry (HRMS).

The aim of this work was to develop and validate an HRMS assay for ST-246 quantification in human plasma in parallel reaction monitoring (PRM) mode and to compare it with the previously reported MRM method.

## 2. Results

### 2.1. Extraction Procedure

To compare the data obtained by the present HRMS method and by the MRM method [16], we used the extraction procedure published in ref. [16]. In brief, we first added an equal volume of methanol to plasma to obtain a fine suspension and then applied 200 μL of an extraction solvent containing an internal standard (IS). The total volume was 300 μL with a final ST-246 concentration dilution of 1:6, relative to plasma concentration. This extraction is a simple one-step procedure without drying and sample reconstitution, including only 1 h of shaking, centrifugation, and the taking of an aliquot for the mass-spectrometric analysis.

### 2.2. Liquid Chromatography Coupled with HRMS (LC-HRMS)

**Figure 1:**
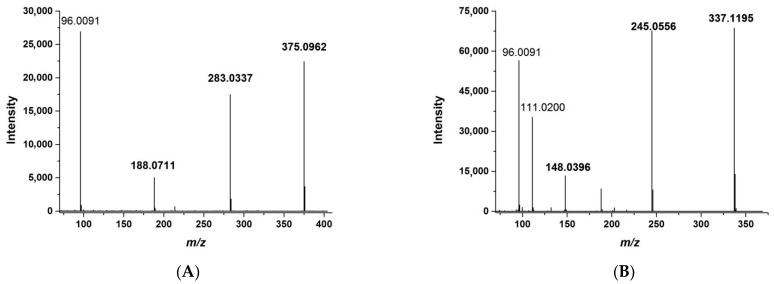
The high-resolution mass spectrum of ST-246 (**A**) and the IS (**B**) registered by the PRM method. For fragment ions selected for the quantification (boldfaced), masses are pointed out.

HRMS allows for the detection of analytes by exact mass. The greater the accuracy of the determined mass, the fewer impurities are detected simultaneously with the analyte. On the one hand, this approach increases selectivity; on the other hand, it reduces the sensitivity of the assay. The best selectivity is provided by a mass tolerance of 1 ppm or less. The use of PRM allows the mass tolerance to be increased to approximately 10 ppm without compromising selectivity. This is achieved by the simultaneous detection of *m*/*z* of both the whole molecule and of the fragment ions, as in the MRM method. At the same time, signals from different fragments could be summed to enhance the total response. For the quantitative assay, we optimized the ST-246 and IS fragmentation by adjusting normalized collision energies; characteristic fragmentation patterns are shown in Figure 1.

**Figure 2:**
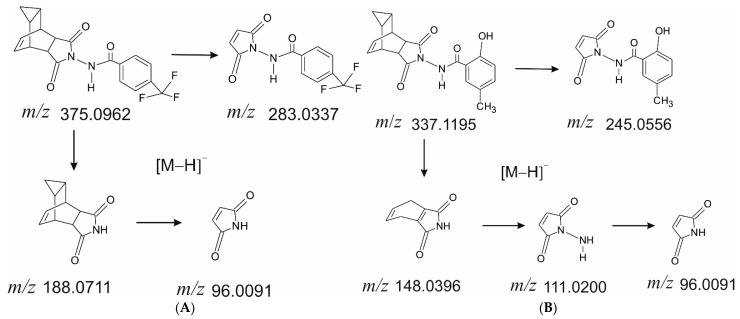
Structures of fragment ions of ST-246 (**A**) and the IS (**B**).

Several fragment ion *m*/*z* values were selected for the detection and quantification: 188.0711, 283.0337, and 375.0962 for ST-246 and 148.0396, 245.0556, and 337.1195 for the IS. The proposed structures of fragment ions calculated from exact mass and the proposed molecular formula as well as the fragmentation pathways are presented in Figure 2.

### 2.3. Linearity

**Figure 3:**
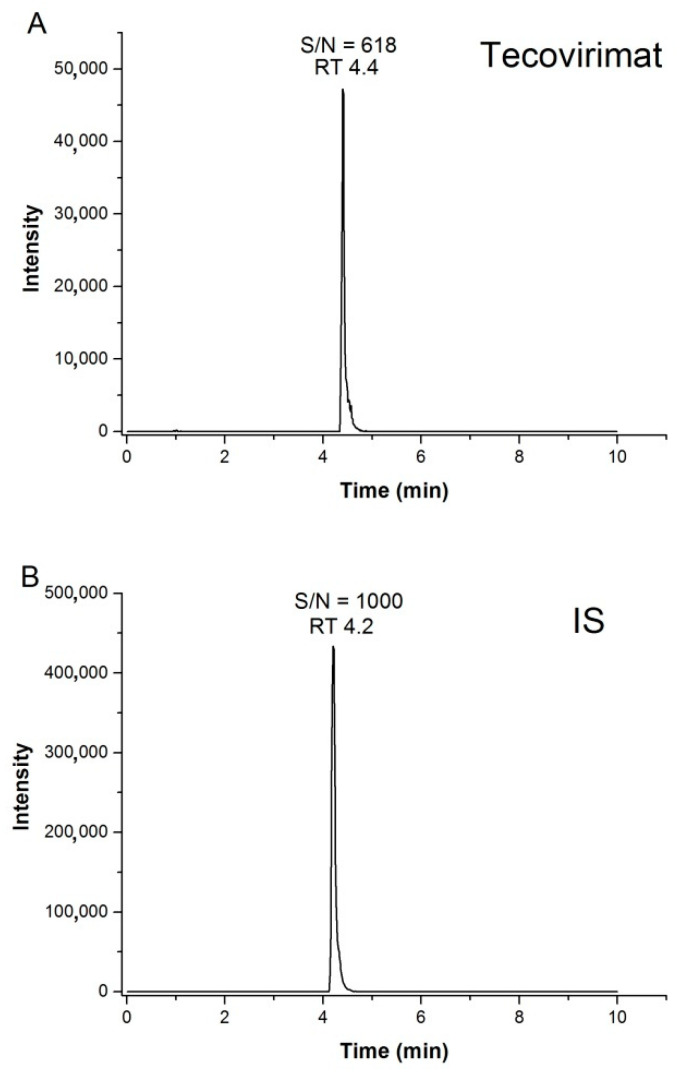
Representative chromatograms of ST-246 at the LOQ (10 ng/mL; panel (**A**)) and of the IS at 30 ng/mL (**B**) with the corresponding retention time (RT) and signal-to-noise (S/N) ratio.

Linearity was evaluated in the range of 10–5000 ng/mL. The correlation between ST-246 concentration and the ratios of the peak areas of ST-246 to those of the IS was described by square regression analysis with 1/x weighting factors. The mean linear regression equation obtained for the proposed method was Y= 0.0133X + 0.0331 with r^2^ = 0.99. Representative chromatograms of ST-246 at the LOQ (10 ng/mL) and of the IS at 30 ng/mL are shown in Figure 3. No signal was detectable in the blank plasma sample.

### 2.4. Accuracy and Precision

To compare the validation parameters of the presented HRMS method with those of tandem mass spectrometry (MS/MS), we chose the same concentration for quality control (QC) levels as reported in ref. [16]: 50 ng/mL for low QC (LQC), 800 ng/mL for medium QC (MQC), 2000 ng/mL for high QC (HQC), and 10 ng/mL for the lower LOQ (LLOQ).

Intra- and inter-day accuracy and precision were evaluated via the analysis of six artificial replicates (artificial samples prepared and analyzed in an identical way in parallel; hereafter: replicates) of each ST-246 QC on 3 consecutive days. The accuracy obtained for QCs ranged from −5.5% to 7.3% and from −2.6 to 2.6% for the intraday and interday validation runs, respectively. The QCs’ precision ranged from 4.7% to 13.6% and from 9.1% to 10.9% for the intraday and interday evaluations, respectively. The accuracy and precision at the LLOQ were within 14.6% for all evaluations (Table 1).

In comparison with the accuracy and precision obtained for the MRM method [16], the present assay shows better accuracy with comparable precision.

### 2.5. Recovery and Matrix Effect

The recovery of ST-246 from plasma was between 87.9% and 100.6%. The recovery of ST-246 was assessed at the QCs’ concentrations via a comparison of the mean measured ST-246 concentration of the extracted samples versus the measured ST-246 concentration of extracted plasma blank samples spiked (after extraction) with ST-246.

The effect of the matrix was assessed via a comparison of the ST-246 concentration in the extracted blank plasma samples spiked (after extraction) with ST-246 versus the same ST-246 concentration in acetonitrile. A slight ion enhancement up to 13.8% was observed (Table 2).

The values of recovery and of matrix effect in the proposed HRMS method were a little higher than those reported for the MRM method [16].

### 2.6. Stability

Plasma samples containing ST-246 were tested for short-term stability at 4 °C, long-term stability at −20 °C, and for stability after three freeze–thaw cycles. No degradation at QC concentrations was observed after three freeze–thaw cycles: 98–105% of nominal ST-246 concentrations was retained. The stability at the LOQ also was within 17.5% deviation of nominal concentration; only percentage relative standard deviation (% RSD) was over 20%. 

The short-term stability experiments at 4 °C in an autosampler revealed good stability within 48 h (Table 3). At −20 °C in a freezer, ST-246 remained stable for 3 months (Table 4).

The determined stability of the validated method was the same as that of the MRM method [16].

### 2.7. A Comparison of the PRM and MRM Methods

**Figure 4:**
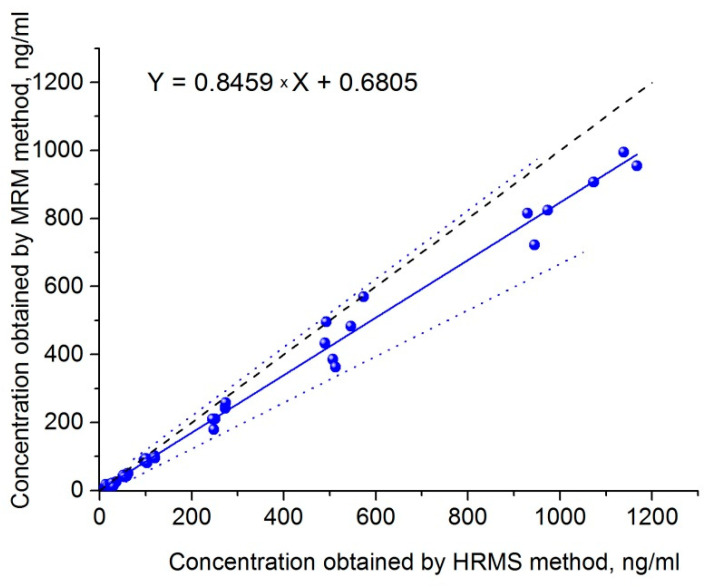
The Passing–Bablok correlation plot. The correlation between ST-246 concentrations in plasma (blue dots), as determined by the validated PRM method (x-axis) and MRM method (y-axis). Solid blue line, regression line; dashed bold line, identity line; dot blue line, confidence interval for the regression line.

**Figure 5:**
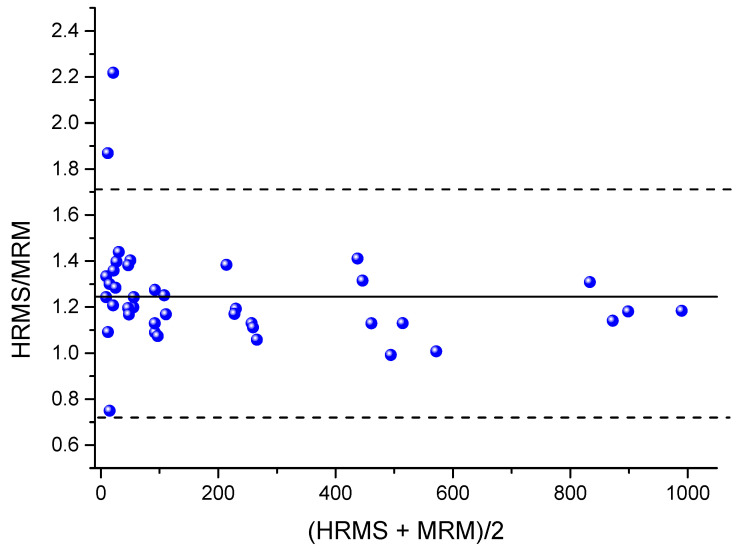
A plot of ratios of concentrations shown by the HRMS method (PRM) to those shown by the MRM method versus the mean sample concentrations revealed by the two methods (blue dots). The solid black line denotes the mean ratio of 1.24, and the dashed lines represent the 95% confidence interval.

To compare the presented PRM method with the MRM method, which was successfully applied to samples of NIOCH-14 in a clinical study [16], the same samples were analyzed by both techniques. The Passing–Bablok plot of correlation between ST-246 concentrations obtained by the MRM and PRM methods is displayed in Figure 4. It was found that higher concentrations were shown by the HRMS method. The correlation was described by means of the following equation: Y = 0.8459X + 0.6805, and Pearson’s correlation coefficient was 0.9956. A plot of ratios of concentrations output by the PRM method to those output by the MRM method versus mean sample concentrations is presented in Figure 5. The mean ratio of concentrations shown by the MRM and PRM methods was 1.24 (95% confidence interval: 0.72 to 1.71), which confirms the conclusion that a higher concentration is output by the PRM method in comparison with the MRM method. 

## 3. Discussion

In comparison with QQQ mass spectrometers, high-resolution mass spectrometers are usually less sensitive due to longer time being spent on every scan to achieve the high resolution instead of accumulating signals. The application of the PRM method to HRMS allows simultaneous detection not only of the whole molecular ion but also of its fragments. Thus, summing of peak areas of different fragments increases the sensitivity of the method owing to the stronger signal in comparison to methods where only the whole molecule is analyzed by HRMS. On the other hand, the PRM method looks like an improved MRM method, simultaneously allowing for the identification of several fragments of a molecule with high resolution and summing them up. In fact, the PRM method is an analog of the MRM method, where several transitions are determined, of which only one transition is used for the quantitative analysis. By contrast, in PRM, all fragments could be used for both quantitative and qualitative analysis at the same time.

During the PRM method development, we determined the structure of fragments of ST-246 and of the IS (Figure 2) for obtaining intense signals in the fragmentation pattern of the molecules. For ST-246 quantification, we chose the whole-molecule ion with *m*/*z* 375.0962 and two fragments with *m*/*z* 188.0711 and 283.0337. Some fragments of ST-246 and of the IS were identical due to similar structures. Therefore, to avoid interference, the fragments with *m*/*z* 148.0396 and 245.0556 of the IS (they are different from ST-246 fragments) were chosen for IS detection along with the IS whole-molecule ion, *m*/*z* 337.1195.

The amplification of the final signal via the summation of signals of several *m*/*z* values made it possible to achieve comparable efficiency of the proposed method relative to MRM methods. In this regard, the HRMS method showed similar accuracy, precision, recovery, and stability during the validation as compared to MRM [16]. Moreover, the linear range of the PRM method proved to be 10 to 5000 ng/mL, whereas in MRM methods, this range is only 50–4000 ng/mL [13,14,15] or 10–2500 ng/mL [16]. 

Our comparison of the MRM and PRM methods applied to the same samples indicates that the concentration shown by the PRM method is approximately 24% higher and closer to the actual one (Figure 5). Because this difference is seen throughout the whole concentration range, it could be caused by applying the different mass spectrometry technique and should be taken into account when choosing an assay for ST-246. Apparently, some substances with *m*/*z* transitions similar to those of ST-246 are also present in our plasma extract. Due to the low selectivity of the MRM method, they could be detected along with ST-246 in the MRM method and thus distort the calibration curve, thereby introducing an error into the measurement. This is evidenced by a decrease in the spread of concentration ratio PRM/MRM with the increasing concentration (Figure 5). By contrast, in the PRM method, such interfering substances are excluded due to higher resolution of *m*/*z* values, thus making the presented method more preferable.

## 4. Material and Methods

### 4.1. Chemicals and Reagents

ST-246 and 2-hydroxy-N-{3,5-dioxo-4-azatetracyclo[5.3.2.0^2.6^.0^8.10^]dodec-11-en-4-yl}-5-methylbenzamide (IS) were synthetized for research proposes with yield >96% and kindly provided by A.Ya. Tikhonov and B.A. Selivanov (N.N. Vorozhtsov Novosibirsk Institute of Organic Chemistry SB RAS, Russia). LC-MS grade acetonitrile was purchased from Biosolve (Dieuze, France). Methanol of HPLC grade was obtained from J. T. Baker (Gliwice, Poland). HPLC grade water for the experiments was produced with a Milli-Q purification system from Millipore Corp. (Bedford, MA, USA). LC-MS grade formic acid was acquired from Sigma-Aldrich (St. Louis, MO, USA). Human plasma was obtained from healthy volunteers with approval by the Human Ethics Committee of the Institute of Chemical Biology and Fundamental Medicine (protocol code 10 of 26 December 2019). The volunteers consisted of people aged 18 years or older with the following inclusion criteria: had no contraindications, were healthy, and had not taken other medications within the last 7 days. Blood sampling from the cubital vein was performed in the morning on an empty stomach after at least 12 h of fasting in 4 mL vacutainer plasma separation tubes. Blood plasma was obtained according to the standard method by centrifugation at 1500× *g* for 10 min using a refrigerated centrifuge at 4 °C.

### 4.2. Stock Solutions, Standards, and QCs

ST-246 and IS stock solutions were prepared in acetonitrile to attain a final concentration of 10 mg/mL. All stock solutions were stored at −22 °C until use.

The ST-246 working solution was made up on the day of analysis via dilution of the ST-246 stock solution in acetonitrile to a final concentration of 100 μg/mL. The IS working solution at a final concentration of 30 ng/mL was prepared in the same way. The working solutions were kept at 4 °C until experiments.

To prepare the calibration standards, at the first step, the ST-246 working solution was serially diluted in acetonitrile to obtain concentrations of 50,000, 25,000, 10,000, 5000, 2500, 1000, 500, 200, 100, 50, and 25 ng/mL. The calibration standards were prepared immediately prior to use by tenfold dilution in plasma to obtain final concentrations of 5000, 2500, 1000, 500, 250, 100, 50, 20, 10, 5, and 2.5 ng/mL. For tenfold dilution in plasma, 5 μL of the ST-246 solution was added to 50 μL of plasma in 1.5 mL tubes. The microtubes were shaken for 30 min at 37 °C and 900 rpm in a TS-100C thermo-shaker (BioSan, Latvia) to ensure full interaction of ST-246 with plasma proteins.

QCs were prepared similarly to calibration standards with final concentrations in plasma of 2000, 800, 50, and 10 ng/mL for HQC, MQC, LQC, and LLOQ, respectively.

### 4.3. Extraction

At the next step, 50 μL of methanol was added, and samples were shaken until a fine suspension was obtained, followed by the addition of 200 μL of the IS in acetonitrile. To extract ST-246, the samples were shaken for 60 min at 40 °C and 900 rpm. After centrifugation at 13,000× *g* for 10 min, 130 μL of the supernatant was transferred into vials for subsequent injection into an ultra-high-performance liquid chromatography (UPLC) system.

### 4.4. LC-HRMS in PRM Mode

A DIONEX UltiMate 3000 HPLC system (Thermo Fisher Scientific, Inc., Waltham, MA, USA) was used coupled to a Q Exactive HF Orbitrap mass spectrometer (Thermo Fisher Scientific, Inc.). The Xcalibur 4.2.47 software (Thermo Fisher Scientific, Inc.) was employed for setting up the analysis and for data management. Chromatographic separation was performed on a ProntoSil-120-3-C18 analytical column (2 × 75 mm, 3 μm) (EcoNova, Russia) with a Zorbax Eclipse XBD-C18 guard column (4.6 × 12.5 mm, 5 μm). The mobile phase was composed of eluent A (water) and eluent B (acetonitrile). The temperatures of the autosampler and column were maintained at 4 and 50 °C, respectively. Chromatographic separation was performed at 300 μL/min in a 10 min run using the following gradient: 2% of buffer B from minute 0 to minute 0.3, 100% of buffer B at 7 min, 2% of buffer B from minute 7.01 to minute 10.

Mass-spectrometric analysis was performed on the Q Exactive HF Orbitrap mass spectrometer (Thermo Fisher Scientific, Inc.). The electric potential was set to 4.2 kV, and the nitrogen sheath and Aux gas flow were 9 and 3 arbitrary units, respectively. The capillary temperature was set to 320 °C. Quantification of ST-246 was performed in PRM mode with negative polarity at a resolution of 45,000 at *m/z* 200. Full scans were acquired with automatic gain control (AGC) 2E4, injection time of 100 ms, and normalized collision energies of 20, 40, and 60 eV. Precursor ions were filtered by the quadrupole using isolation window of 4 *m/z* (±2 *m/z* of selected *m/z*). The inclusion list consisted of two lines with the following settings: *m/z* 375.0962 for ST-246 and *m/z* 337.1194 for IS. Mass tolerance for both compounds was set to 10 ppm. Several fragment ion *m/z* values were selected for detection and quantification: 188.0711, 283.0337, and 375.0962 for ST-246 and 148.0396, 245.0556, and 337.1195 for the IS.

### 4.5. Validation Procedures

Linearity, accuracy, precision, recovery, matrix effect, and sample stability were examined for validation. The method was validated in accordance with European Medicines Agency (EMA) guidelines [17].

#### 4.5.1. The Calibration Curve and LLOQ

To determine the linearity of the newly developed method, the calibration curve was constructed by means of eight concentrations of ST-246 by plotting peak area ratios ST-246/IS against ST-246 concentrations in the spiked plasma standards. The lowest standard in the calibration curve with an S/N ratio >10 was assumed to be the LLOQ.

#### 4.5.2. Accuracy and Precision

Intraday and interday accuracy and precision were assessed by analysis of replicates of samples at LOQ and QC concentrations. For determining intraday bias and accuracy, the QCs and LOQ samples (n = 12) were assayed within 1 day. For interday evaluation, six replicates of QCs and of LOQ were analyzed on 3 consecutive days. Accuracy was expressed as percentage deviation of the mean from a nominal concentration. Precision was calculated as percentage RSD (% RSD). Acceptable tolerance for QCs was set to ±15%; for LOQ, it had to be within ±20%.

#### 4.5.3. Recovery and Matrix Effect

Recovery was calculated via a comparison of measured concentrations of QCs extracted from plasma to those of QCs added to the plasma extract of blank samples. Matrix effect was calculated as a ratio of the QCs added into the plasma extract of blank samples to the same concentration in acetonitrile.

#### 4.5.4. Stability

ST-246 in plasma stability was evaluated after three freeze–thaw cycles. Short-term stability of ST-246 after extraction was evaluated after storage at 4 °C for 48 h. Long-term stability of ST-246 stored at −20 °C in plasma was evaluated for 3 months. All stability tests were performed on QCs and LOQ samples in four replicates.

## 5. Conclusions

An accurate and sensitive HRMS assay for ST-246 quantification in human blood plasma was developed and fully validated in PRM mode. Fragment ions of ST-246 were identified by their exact mass and were used together to improve both the sensitivity and selectivity of the presented method. The precision, accuracy, and specificity of the method are within acceptable ranges according to EMA recommendations in the 10–5000 ng/mL concentration range. ST-246 was found to be stable in plasma samples at −20 °C for at least 3 months and up to 48 h after extraction at 4 °C. A comparison of the MRM and PRM methods indicates that the concentration output by the PRM method is approximately 24% higher, which should be taken into account when choosing an assay for ST-246. Nevertheless, the high selectivity with good sensitivity, as compared with traditional MRM methods, makes this approach attractive for research and clinical use.

## Figures and Tables

**Table 1:** Intraday and interday accuracy and precision.

Concentration, ng/mL	Intraday						Interday	
	1st Day		2nd Day		3rd Day			
	% Bias	% RSD	% Bias	% RSD	% Bias	% RSD	% Bias	% RSD
10	−12.5	7.1	2.7	14.6	−8.0	9.0	−7.6	10.9
50	2.9	10.4	7.3	5.2	−1.3	6.4	2.6	9.1
800	2.3	12.3	0.0	4.7	−3.8	5.4	0.7	10.3
2000	1.2	13.6	−4.4	10.7	−5.5	6.8	−2.6	10.9

**Table 2:** Recovery and matrix effect.

Concentration, ng/mL	Recovery (%)	Matrix Effect (%)
10	98.1	101.9
50	100.6	99.4
800	94.1	106.3
2000	87.9	113.8

**Table 3:** Stability of ST-246 in plasma after 48 h storage at 4 °C.

Concentration, ng/mL	0 h		5 h		24 h		48 h	
	% Bias	% RSD	% Bias	% RSD	% Bias	% RSD	% Bias	% RSD
10	−15.1	4.2	−0.8	6.0	−1.3	7.0	6.3	12.7
50	−4.2	7.5	1.6	3.8	6.3	6.6	10.5	10.3
800	−5.5	5.8	−2.2	5.0	1.8	6.3	0.8	6.5
2000	−10.6	4.2	−0.4	4.7	1.3	3.7	−6.0	9.0

**Table 4:** Stability of ST-246 in plasma after storage at −20 °C for 3 months.

Concentration, ng/mL	0 Day		13 Days		44 Days		90 Days	
	% Bias	% RSD	% Bias	% RSD	% Bias	% RSD	% Bias	% RSD
10	8.3	8.9	−15.1	11.1	−17.1	10.2	1.9	13.0
50	11.6	10.0	−10.2	10.2	−12.7	4.4	14.0	8.0
800	−2.3	14.5	−5.6	8.2	−7.7	4.4	13.0	7.7
2000	−7.0	5.7	−9.2	9.7	−14.5	5.7	7.4	8.4

## Data Availability

Data are available on request, owing to privacy and ethical restrictions.
